# Electron-Level Mechanistic Insights into Ce Doping for Enhanced Efficiency Degradation of Bisphenol A under Visible Light Irradiation

**DOI:** 10.3390/nano12081382

**Published:** 2022-04-18

**Authors:** Qi Zeng, Chu-Ya Wang, Bo-Xing Xu, Jianyu Han, Xin Fang, Guangcan Zhu

**Affiliations:** School of Energy and Environment, Southeast University, Nanjing 210096, China; zeng_qi@seu.edu.cn (Q.Z.); 213190216@seu.edu.cn (B.-X.X.); hanjy@seu.edu.cn (J.H.); fxin@seu.edu.cn (X.F.)

**Keywords:** bismuth oxybromide (BiOBr), Ce-doping modification, doping energy level, visible light, bisphenol A (BPA)

## Abstract

Bismuth oxybromide (BiOBr), with its special layered structure, is known to have potential as a visible-light-driven photocatalyst. However, the rapid recombination and short lifetime of the photogenerated carriers of BiOBr restrict its photocatalytic efficiency for the degradation of organic pollutants. Given the similar ionic size of Ce and Bi, Ce atoms might be easily introduced into the crystal of BiOBr to tailor its band structure. In this study, Ce doped BiOBr (Ce-BiOBr) samples with different percentages of Ce contents were prepared via a hydrothermal method. The intrinsic photocatalytic efficiency of Ce_0.2_-BiOBr for the degradation of bisphenol A (BPA) was 3.66 times higher than that of pristine BiOBr under visible light irradiation. The mechanism of Ce-doping modification for the enhanced photocatalytic performance was demonstrated based on a series of experiments and DFT calculation. The narrowed bandgap, the enhanced charge separation efficiency and Ce-doping energy level contributed to the remarkable photocatalytic performance of Ce-BiOBr.

## 1. Introduction

Semiconductor photocatalysis has received increasing attention in recent decades due to its potential uses in wastewater treatment and energy conversion [1] because it can directly harvest solar energy and convert it into chemical energy. Among the various semiconductor photocatalysts to date, titanium dioxide (TiO_2_) has received the most attention due to its strong oxidizing properties, low cost and high stability [2]. However, the large intrinsic band gap (>3.2 eV) of TiO_2_ leads to an inability to directly absorb visible light [3]. Namely, TiO_2_ can only harvest ultraviolet (UV) light, indicating its poor utilization efficiency of visible light. The rapid recombination efficiency of photogenerated electrons (e^−^) and holes (h^+^) also limits its application [4]. Previous works confirm that doping modification is a promising strategy to solve this problem. For example, Cl-doped rutile TiO_2_ possessed a strong response towards visible light irradiation and a higher charge separation efficiency [4].

Apart from modified TiO_2_, bismuth oxybromide (BiOBr) is also a potential visible-light-driven photocatalyst with a highly anisotropic layered structure. The specific layered structure consists of [Bi_2_O_2_]^2+^ and [Br_2_]^2−^ layers, which are stacked in the BiOBr crystal [5]. Therefore, there is a strong internal static field perpendicular to each layer, which can promote the separation of e^−^ and h^+^ to some extent [6]. Previous studies have verified potential applications of BiOBr for pollutant removal, nitrogen fixation, disinfection, and CO_2_ reduction [7,8,9,10]. However, the utilization efficiency of the sample to visible light is still desired to be enhanced for practical applications. Given that the optical property of a photocatalyst is highly related to its band structures, doping is one of the most effective strategies for its regulation [11]. It has been reported that the photocatalytic performance of semiconductor photocatalysts can be improved by using doping-selected cations as e^−^ capture agents, which can efficiently suppress the recombination efficiency of charge carriers. For example, Wang et al. confirmed that the Bi-doping modification can retard the recombination of photogenerated e^−^/h^+^ in BiOBr [12]. Moreover, B-doping modification can efficiently accelerate the charge carries separation of BiOBr, and thus results in an enhanced photocatalytic performance for the degradation of rhodamine B (RhB) under visible light irradiation [8].

Cerium (Ce) is a typical rare earth element and is commonly used as a dopant of various photocatalysts, including Ce/ZnO [13], Ce/MnO_2_ [14], and Ce/SnO_2_ [15]. Due to the similar ionic size of Ce (1.03 Å) and Bi (1.03 Å) atoms, Ce atom might be easily introduced into the crystal structure of BiOBr as a dopant. Until now, studies of the Ce-doping modification on BiOBr have been very limited [16]. Ce-doped BiOBr micro-sheets were obtained through a hydrothermal procedure and showed good performance for the degradation of RhB under visible irradiation. However, RhB is a typical organic dye, which has strong dye-sensitization during light irradiation. Given that most organic pollutions are non-dye molecules, the intrinsic photocatalytic efficiency of BiOBr is still desired to enhance. Therefore, nanometer-sized Ce-doped BiOBr materials need to be synthesized and the effects of the doped Ce element and the mechanism of the photocatalytic process still remain to be elucidated.

In this study, a series of Ce-doped BiOBr (noted as Ce-BiOBr) samples were synthesized through a mild hydrothermal method. Characterizations were carried out systematically to explore the effects of Ce doping on the morphology, optical absorption, and photoelectrochemical and catalytic degradation properties. Bisphenol A (BPA), a typical non-dye organic molecule, was the target pollutant to investigate the intrinsic photocatalytic performance of the as-prepared products under visible irradiation. Kinetic models for the degradation of BPA over the as-prepared products were established and the main active species was confirmed. More importantly, the effects of the crystal structure and electronic state of BiOBr caused by Ce-doping modification were analyzed via a density functional theory (DFT) calculation. The results of this study have significant implications for the design and application of BiOBr-based photocatalysts for wastewater treatment.

## 2. Materials and Methods

### 2.1. Materials and Chemicals

In this work, the anhydrous ethanol was purchased from Sinopharm Chemical Reagent Co., Ltd. (Shanghai, China). All other chemicals including bismuth nitrate pentahydrate (Bi(NO_3_)_3_∙5H_2_O), sodium bromide (NaBr), cerium nitrate hexahydrate (Ce(NO_3_)_3_∙6H_2_O), ethylene glycol (C_2_H_6_O_2_), and BPA (C_15_H_16_O_2_) were purchased from Aladdin Reagent Co., Ltd. (Shanghai, China). All reagents were of analytical grade and used without further purification.

### 2.2. Synthesis of Ce-BiOBr nanoflakes

The Ce-BiOBr nanoflakes were prepared through a mild hydrothermal procedure with different Bi/Ce ratios. In a typical procedure, both Bi(NO_3_)_3_∙5H_2_O (4 mmol) and a specific amount of Ce(NO_3_)_3_∙6H_2_O (0.1, 0.4, 0.8, and 2 mmol) were added to 30 mL of ethylene glycol (EG) and sonicated for 5 min. In this way, a homogeneous EG solution of Bi^3+^ and Ce^3+^ was obtained. Meanwhile, NaBr (4 mmol) was dissolved in 40 mL of deionized water. The aforementioned two solutions were mixed and transferred to a 100-mL Teflon reaction kettle and stirred for 5 min at a speed of 400 rpm. After that, the mixture was heated at 160 °C for 12 h and then cooled down to temperature naturally. The resulting products were collected centrifugally and washed three times using distilled water and anhydrous ethanol, respectively. Finally, the product was dried in a vacuum at 80 °C for 12 h. The samples were noted as Ce_0.05_-BiOBr, Ce_0.1_-BiOBr, Ce _0.2_-BiOBr, and Ce _0.5_-BiOBr according to their molar ratios of Bi:Ce (1:0.05, 1:0.1, 1: 0.2, and 1:0.5, respectively). Similarly, BiOBr was synthesized through the same procedure but without the utilization of Ce(NO_3_)_3_∙6H_2_O.

### 2.3. Physicochemical Characterization

For characterization, X-ray diffraction (XRD) measurements of the samples were performed on a smart X-ray diffractometer (Rigaku, Japan) using Cu Kα radiation, with a scanning rate of 10° S^−1^ and 2θ range from 10 to 80°. The morphologies of the samples were determined through scanning electron microscopy (SEM; Gemini 300; Zeiss, Jena, Germany). Transmission electron microscopy (TEM), high-resolution TEM (HRTEM), scanning transmission electron microscopy (STEM), and energy dispersive spectrometry (EDS) mapping measurements were obtained with an FEI Talos F200s instrument (Thermo Fisher Scientific, USA). The elemental composition was obtained through X-ray photoelectron spectroscopy (XPS; Thermo Fisher Scientific, Waltham, MA, USA). The optical absorbance was determined using a UV-Vis spectrophotometer (PE Lambda 950; Perkin Elmer, Waltham, MA, USA). The steady state photoluminescence (PL) emission spectra and time-resolved fluorescence spectra were obtained using a steady state and transient state fluorescence spectrometer (FLS1000; Edinburgh Instruments, Livingston, UK). The existence of free radicals was performed on electron paramagnetic resonance (EPR) spectrometer (Bruker EMXplus-6/1, Bruker, Germany).

### 2.4. Electrochemical Characterization

All electrochemical characterization procedures were performed using an electrochemical workstation (CHI760E; CH Instrument Co., Shanghai, China) containing a three-electrode system. The reference and counter electrodes were Ag/AgCl (KCl, 3 M) and a Pt wire, respectively. The materials were deposited on a glassy carbon electrode, which served as the working electrode for electrochemical impedance spectroscopy (EIS) measurements. A K_3_[Fe(CN)_6_] and K_4_[Fe(CN)_6_] aqueous solution with a concentration of 0.5 M was used as the electrolyte for the EIS analysis. Photocurrent response tests were performed in a 0.1 M Na_2_SO_4_ electrolyte solution, with a bias voltage of 0.2 V for 600 s, in which the F-doped SnO_2_ (FTO) glass was used as the working electrode.

### 2.5. Photocatalytic Degradation of BPA

The photocatalytic degradation of samples of BPA was performed in a jacketed beaker maintained at a constant temperature. The light source was a 500W Xenon lamp (CHF-XM500; Beijing Perfectlight, Beijing, China) with a 420-nm cutoff filter. Typically, 10 mg of photocatalyst was distributed into 40 mL BPA solution with a concentration of 10 mg/L, and then sonicated for 1 min to achieve a full dissolution. The mixture was stirred for 30 min at a speed of 400 rpm in the dark to achieve an adsorption equilibrium between the pollutants and photocatalyst. Each sample was acquired at a given time and the concentration of BPA was analyzed using a high-performance liquid chromatography (HPLC; Primaide; Hitachi, Tokyo, Japan) with a C18 column. The temperature of the column oven was set to 40 °C. The flow rate was maintained at 0.5 mL/min with a mobile phase of 50% eluent A (acetonitrile) and 50% B (water containing 1‰ formic acid). The injected volume was 10 μL and retention time was set to 8 min.

### 2.6. Theoretical Calculation

The DFT calculations were carried out using the Vienna Ab-initio Simulation Package (VASP) [17,18] with the frozen-core all-electron projector-augment-wave (PAW) [19] method. The generalized gradient approximations (GGA) of Perdew–Burke–Ernzerhof (PBE) were adopted to describe the exchange and correlation potential [20]. The cutoff energy for the plane-wave basis set was set to 450 eV. A 2 × 1 × 1 BiOBr supercell was used. Geometry optimizations were performed until the forces on each ion were reduced below 0.01 eV/Å, and 1 × 3 × 3 k-point sampling of the Brillouin zone was applied [21]. The resulting structures were then used to calculate the electronic structures, and the k-point sampling was increased to 3 × 5 × 5. The formation energies (*E*_f_) of Ce doping were calculated by the following formulas:

(1) $$E_{f}=E_{d}-E_{p}-\mu_{\mathrm{Ce}}+\mu_{r}$$

where *E*_d_ and *E*_p_ are the total energies of Ce-doped and pure BiOBr, respectively. *μ*_Ce_ is the chemical potential of the Ce atom, and *μ*_r_ is the chemical potential of the replaced atom (Bi, O, and Br).

## 3. Results and Discussion

### 3.1. Structure and Morphology of the Catalysts

**Figure 1:**
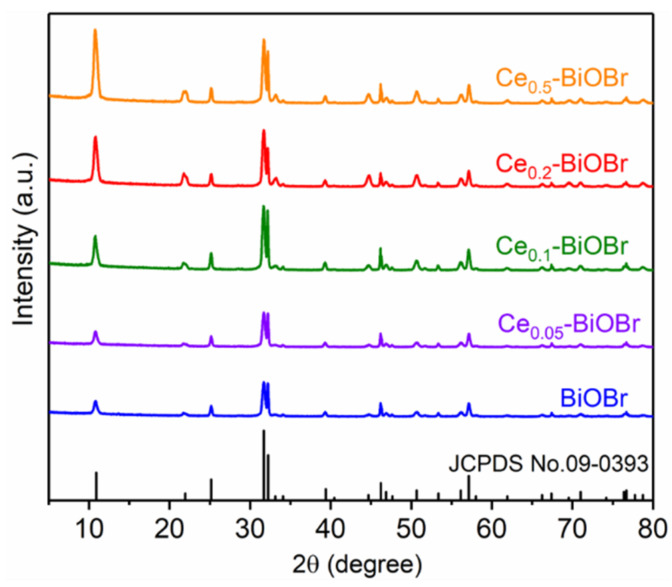
XRD spectra of samples.

The phase identification of the as-prepared product was determined using XRD. Figure 1 shows the diffraction peaks of these products. For all samples, the diffraction peaks could be indexed to BiOBr (JCPDS No. 09-0393), demonstrating that Ce-doping modification did not change the crystal structure of BiOBr framework. And all these peaks showed sharp patterns and no impurity peaks were noticeable, indicating the high crystallinity and purity of the samples. The diffraction peaks at 10.9° could be ascribed to the (001) crystal planes of BiOBr. It was attributed to the stacking structure among [Br-Bi-O-Bi-O-Br] layers along the c-axis. Another peak at 32.22° belonged to (110) crystal planes of BiOBr, which was perpendicular to (001) plane. And the (001) peak became stronger with the increase in Ce doping amount, showing that Ce doping might have an effect on the crystal growth. Moreover, there were no diffraction peaks associated with Ce elements, indicating that the doped Ce was highly dispersed in the host crystal of BiOBr and possessed a homogeneous distribution.

**Figure 2:**
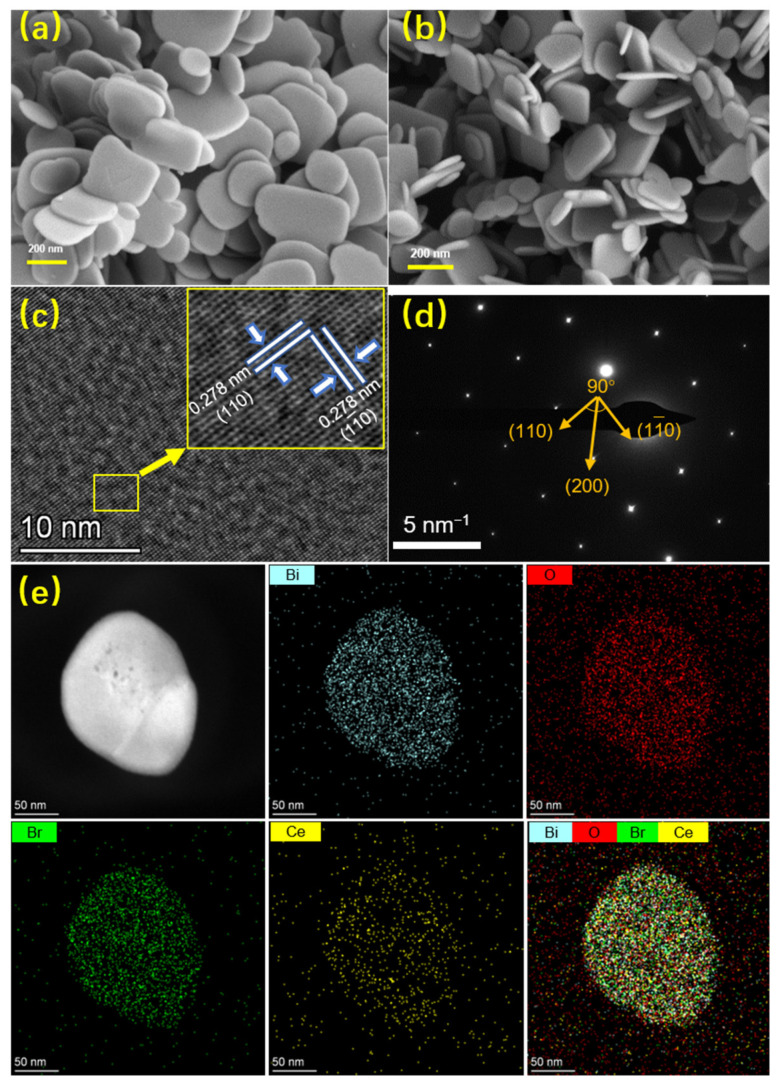
SEM images of (**a**) BiOBr and (**b**) Ce_0.2_-BiOBr. (**c**) HRTEM, (**d**) SAED, (**e**) STEM and corresponding EDS mapping images of Ce_0.2_-BiOBr.

The morphology of the as-prepared product was observed from the SEM and TEM images shown in Figure 2a,b. Both BiOBr and Ce-BiOBr possessed a two-dimensional flake-like structure with a mean size of 200 nm and thickness of about 10 nm. It was demonstrated that the morphology of the samples was a nanosheet and not affected by Ce-doping modification. The HRTEM image of Ce_0.2_-BiOBr (Figure 2c) displayed a clear and continuous lattice fringe with a distance of 0.278 nm, corresponding to the (110) and (1–10) planes of tetragonal BiOBr. This was also confirmed by the selected area electron diffraction (SAED) pattern (Figure 2d). It shows that the angle between the crystal planes of (110) and (200) was 45° and the direction perpendicular to both the (110) and (1–10) facets was (001) crystal axis. Therefore, the (001) facet of as-prepared product was highly exposed. Moreover, the elemental composition of Ce_0.2_-BiOBr was verified using EDS mapping (Figure 2e). The results show that Ce_0.2_-BiOBr consisted of Bi, O, Br, and Ce elements, verifying the presence of Ce element.

**Figure 3:**
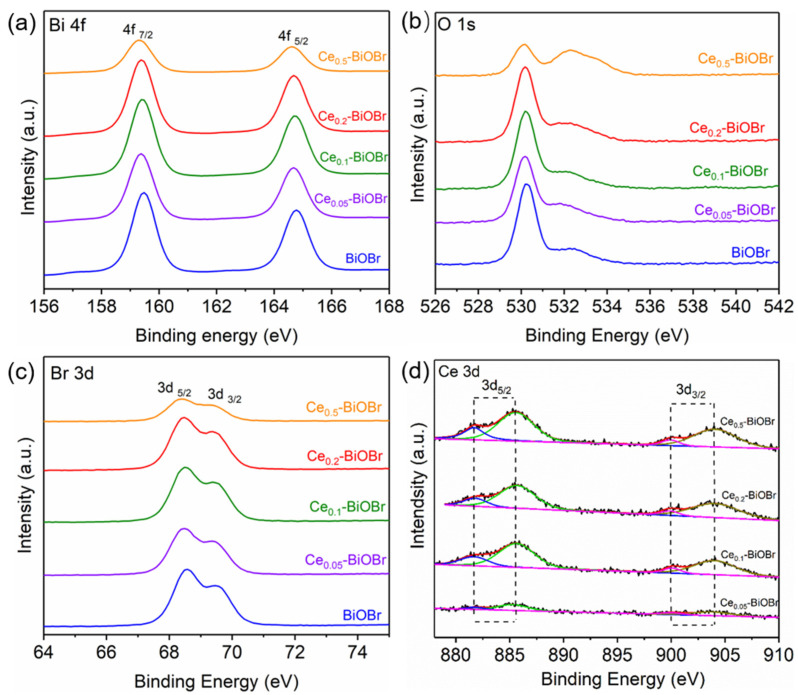
(**a**) Bi 4f, (**b**) O 1s, (**c**) Br 2d and (**d**) Ce 3d spectra of XPS tests.

The surface composition and chemical state of BiOBr and Ce-BiOBr were further characterized using XPS. The charging effects were corrected by using the C 1s peak as reference at binding energy of 284.6 eV. The survey spectra (Figure S1) show three photoelectron lines at 68.17, 159.08, and 530.32 eV that were attributable to Br 3d, Bi 4f, and O 1s signals, respectively. The signal peak of Ce 3d was very weak in the survey spectra due to its low content. The peaks at 159.47 and 164.76 eV corresponded to Bi 4f_7/2_ and Bi 4f_5/2_, respectively [22] (Figure 3a). For O element, the O 1s spectra (Figure 3b) shows an obvious peak at 530.28 eV, which belongs to the lattice oxygen. The peak at 532.52 eV could be ascribed to adsorbed oxygen species at the vacancy sites [23], indicating that oxygen vacancies was introduced into the crystal of the as-prepared products. Some oxygen vacancies might be introduced as a common lattice defect, which was confirmed by the results of XPS spectra of O 1s. Figure S2 shows that all these Ce-BiOBr samples and BiOBr sample contained oxygen vacancies. In the Br 3d spectra (Figure 3c), the binding energies at 68.57 and 69.57 eV corresponded to Br 3d_5/2_ and Br 3d_3/2_ [24], respectively. The two peaks at 884.92 and 904.04 eV in Figure 3d belong to Ce 3d_5/2_ and Ce 3d_3/2_, respectively [15,25], which was due to the 3d spin-orbit coupling effect. The strong satellite peaks locating at around 885.7 and 904.2 eV are due to the bonding of Ce^3+^ with BiOBr. The present Ce 3d indicates the co-existence of Ce^3+^ and Ce^4+^ bonding states [26,27], and the Ce^3+^/Ce^4+^ redox transformation may induce the generation of oxygen vacancies [28], thus affecting the shoulder peak in O 1s spectra. Moreover, as is shown in Figure 3a–c, the peak signals of Bi, O, and Br shifted towards lower binding energy after Ce-doping modification, and the shifts were more obvious when more Ce atoms was doped into the BiOBr crystal. Given that the Ce atoms were likely to dope at the Bi sites in the BiOBr crystal and the electronegativity of Ce atoms were lower than Bi atoms, the doped Ce element could increase the electron densities of Bi, O, and Br atoms, which resulted in the shift of XPS signals towards lower binding energy.

According to the aforementioned results, the presence of Ce element was confirmed based on the EDS mapping result. However, no other impurities were observed in SEM images of Ce-BiOBr, while the XRD patterns did not show any diffraction peak corresponding to Ce or Ce ions either. This might be a result of the low concentration of Ce dopant which did not form a heterojunction with BiOBr. Moreover, the weak XPS signal peak of Ce 3d in the survey spectra also verified its low content, and the shift of XPS signals towards lower binding energy indicates that the doped Ce was uniformly distributed in the BiOBr host crystal. Namely, the doped Ce element was highly dispersed in BiOBr crystal and probably replaced some Bi atoms rather than forming heterojunctions, which was further demonstrated through DFT calculation in the following part.

### 3.2. Band Structures and Photoelectrochemical Properties

**Figure 4:**
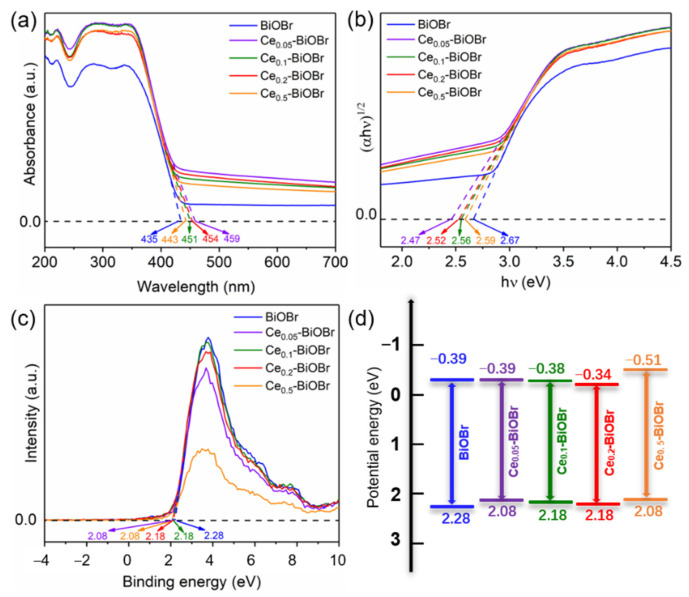
(**a**) UV-Vis DRS, (**b**) Tauc plots, (**c**) valence band and (**d**) band structure diagrams of BiOBr and Ce-BiOBr.

To explore the light absorption properties of the samples, UV-Visible diffuse reflectance spectra (UV-Vis DRS) and band structures of the products were obtained, which is shown in Figure 4. The absorption edge of Ce_0.05_-BiOBr, Ce_0.1_-BiOBr, Ce_0.2_-BiOBr, and Ce_0.5_-BiOBr were measured as 459, 451, 454, and 443 nm, respectively, while the absorption edge of BiOBr was 435 nm. This result confirms that Ce-doping modification resulted in the red-shift of the absorption edge of BiOBr. Therefore, a higher absorption efficiency of visible light for Ce-BiOBr was obtained after Ce-doping modification. The increase in absorption in the UV-Vis irradiation range could be connected with the band gap of the products [5]. It was considered that BiOBr was an indirect semiconductor, and the values of band gap could be estimated by the Tauc curves [5,7], which could be calculated using the following equation [15,29]:

(2) $$\alpha h\nu =A{\left(h\nu -E_{g}\right)}^{n/2}$$

where α, hν, A and $E_{g}$ are the absorption coefficient, photon energy, a constant and the band gap, respectively. The n value is 4 for BiOBr as a typical indirect band gap semiconductor.

In order to find the intercept on X-axis, the linear portion of curve between (*αhv*)^1/2^ and (*hv*) was extrapolated which measures the value of band gap as represented in Figure 4b. The measured band gaps of BiOBr, Ce_0.05_-BiOBr, Ce_0.1_-BiOBr, Ce_0.2_-BiOBr, and Ce_0.5_-BiOBr were 2.67, 2.47, 2.56, 2.52, and 2.47 eV, respectively. Moreover, the potential energy of the valence band maximum (VBM) was measured using the XPS VB spectra (Figure 4c) and specific values of BiOBr, Ce_0.05_-BiOBr, Ce_0.1_-BiOBr, Ce_0.2_-BiOBr, and Ce_0.5_-BiOBr were 2.28, 2.08, 2.18, 2.18, and 2.08 eV. Then the potential energy of the conduction band minimum (CBM) could be calculated by the results of Tauc curves and XPS VB spectra and values of BiOBr, Ce_0.05_-BiOBr, Ce_0.1_-BiOBr, Ce_0.2_-BiOBr, and Ce_0.5_-BiOBr were −0.39, −0.39, −0.38, −0.34, and −0.51 eV. According to the aforementioned results, the band structures of each sample were shown in Figure 4d. These results demonstrated that Ce doping reduced the band gap of the samples, and thus improved the visible light absorption of the sample.

**Figure 5:**
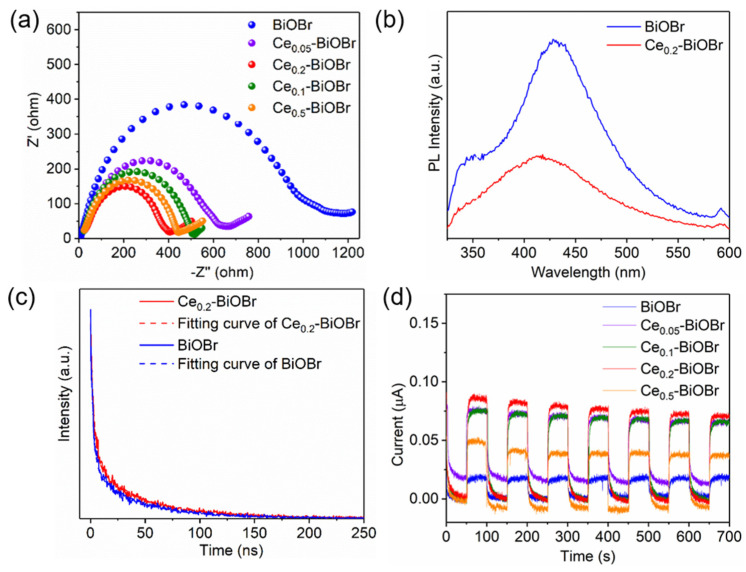
(**a**) EIS tests, (**b**) steady state photoluminescence (PL) emission spectra, (**c**) time-resolved fluorescence spectra and (**d**) transient photocurrent response tests of BiOBr and Ce-BiOBr.

To further explore the effects of the intrinsic electrochemical activity of the catalyst, the efficiency of charge carrier separation and transportation of the photocatalysts were investigated using EIS spectra [30]. The smaller radius of the curvature indicates the smaller impedance of the material. In Figure 5a, the radius of the curvature followed the order of BiOBr > Ce_0.05_-BiOBr > Ce_0.1_-BiOBr > Ce_0.5_-BiOBr > Ce_0.2_-BiOBr, indicating that Ce_0.2_-BiOBr had the lowest impedance, which was 60.3% lower than that of BiOBr. This result confirms that the doped Ce decreased the resistance of BiOBr. To further investigate the recombination efficiency of e^−^/h^+^ pairs in the BiOBr lattice, PL spectra analysis was performed with an excitation wavelength of 310 nm (Figure 5b) [31]. A weaker PL intensity reflects a lower recombination rate of e^−^/h^+^ pairs [32]. The PL emission intensity of BiOBr was stronger than that of Ce_0.2_-BiOBr, indicating that BiOBr had a higher recombination efficiency of e^−^/h^+^ pairs.

Moreover, the time-resolved fluorescence spectra (Figure 5c) were fitted by a biexponential model according to the following equation [33]:

(3) R(t)=B1e−tτ1+B2e−tτ2

where *B*_n_ and *τ_n_* (*n* = 1, 2) are the pre-exponential factor and lifetime in the different processes, respectively. The fits of the fluorescence decay trace of the BiOBr and Ce-BiOBr required double exponential functions to yield an acceptable confidence factor ($x^{2}\approx 1$). Table 1 shows the detailed fitting parameters. The intensity-weighted average lifetime (*τ*), i.e., the mean time delay of photon emission after the picosecond laser pulse, was calculated according to following equation [34]:

(4) $$\tau =\frac{\sum B_{i}\tau_{i}^{2}}{\sum B_{i}\tau_{i}}$$

where $B_{i}$ represents the fractional weights of the various decay time components $\tau_{i}$ of the multi-exponential fitting. The results showed that the lifetimes of the photogenerated carries in BiOBr and Ce_0.2_-BiOBr were 39.82 and 43.78 ns, showing that Ce-doping modification extended the lifetime of charge carries in BiOBr by about 10%. This could be attributed to the inhibited recombination of charges, i.e., more effective separation of e^−^ and h^+^ [35].

Given that the photocurrent density was affected by the charge separation and transfer performance, transient photocurrent response vs time (*i* − *t*) tests were conducted at a constant potential of 0.2 V under periodic illumination (50 s for light on and 50 s for light off). The immediate response to the light on−off cycles implies the appropriate sample fabrication for improvement of charge separation and transfer [36]. As is shown in Figure 5d, the photocurrent response intensity of these samples followed the order of Ce_0.2_-BiOBr > Ce_0.1_-BiOBr > Ce_0.05_-BiOBr > Ce_0.5_-BiOBr > BiOBr, indicating that Ce_0.2_-BiOBr possessed superior electrochemical properties compared to bare BiOBr.

### 3.3. Photocatalytic Degradation of BPA

**Figure 6:**
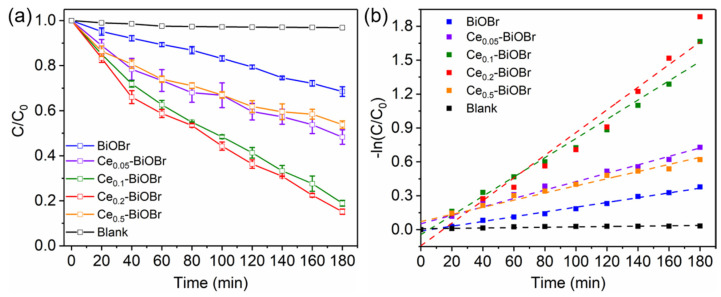
(**a**) Photocatalytic degradation of BPA under 420 nm xenon lamp irradiation and (**b**) corresponding kinetics constant curves.

Photocatalytic degradation of the samples was evaluated through the degradation of BPA, and the results are shown in Figure 6. The result of the blank test determined that the degradation of BPA without photocatalysts under visible light irradiation was negligible. As is shown in Figure 6a, the degradation efficiencies of BiOBr, Ce_0.05_-BiOBr, Ce_0.1_-BiOBr, Ce_0.2_-BiOBr, and Ce_0.5_-BiOBr were 32%, 52%, 81%, 85%, and 46%, respectively. To quantitatively compare photocatalytic properties among these samples, the corresponding kinetic constants were calculated by fitting the experimental degradation curves to the Langmuir-Hinshelwood model. Due to low concentration of the reactant, the following pseudo first-order kinetics equation was used [37]:

(5) $$-\ln\left(C_{t}/C_{0}\right)=kt$$

where *C*_0_ is the initial concentration, *C*_t_ is the concentration at the given time *t*, and *k* is the kinetic constant. The *k* of BiOBr, Ce_0.05_-BiOBr, Ce_0.1_-BiOBr, Ce_0.2_-BiOBr, and Ce_0.5_-BiOBr were calculated as 0.21 × 10^−2^, 0.37 × 10^−2^, 0.85 × 10^−2^, 1.00 × 10^−2^, and 0.32 × 10^−2^ min^−1^, respectively (Figure 6b).

In addition, the BET tests indicated that Ce doping had a slight effect on the surface area of the products (Figure S3). The surface-area-normalized kinetic constants of BiOBr, Ce_0.05_-BiOBr, Ce_0.1_-BiOBr, Ce_0.2_-BiOBr, and Ce_0.5_-BiOBr were 0.32, 0.36, 0.85, 1.17, and 0.59 mg∙min^−1^∙m^−2^ respectively, which eliminated the differences in the exposure of active sites. Namely, the intrinsic photocatalytic activity of Ce_0.2_-BiOBr was 3.66 times higher than that of BiOBr. Moreover, the stability of Ce_0.2_-BiOBr during the photocatalytic degradation of BPA under visible light irradiation is shown in Figure S4. Approximately 80.80% of the photocatalytic activity of Ce_0.2_-BiOBr was retained after five cycles of BPA degradation, and the crystal structure of the Ce-doped BiOBr also retained after five cycles (Figures S5–S7 and Table S1), which confirm its high stability.

As is mentioned above, all these Ce-BiOBr samples and BiOBr sample contained oxygen vacancies in the crystal. However, the photocatalytic efficiency of Ce-BiOBr was substantially higher than that of BiOBr, indicating that the impact of oxygen vacancy was very limited. Therefore, the improvement of photocatalytic degradation performance of samples could be mainly ascribed to the improved visible light absorption and the enhanced charge carries separation efficiency rather than the presence of oxygen vacancies.

### 3.4. Mechanism of BPA Photocatalytic Degradation

**Figure 7:**
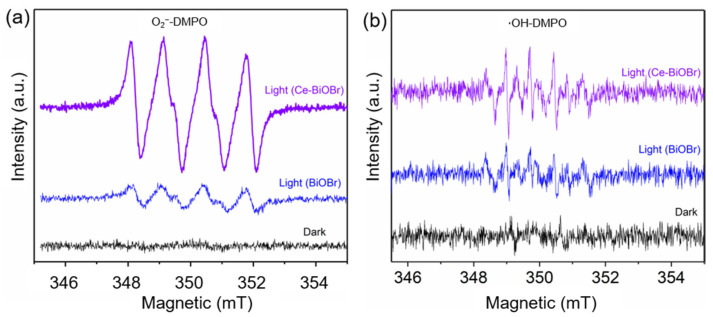
EPR spectra of (**a**) ∙O_2_^−^ and (**b**) ∙OH.

EPR tests were also conducted to determine the existence of ∙O_2_^−^, ∙OH, and h^+^. No EPR signal was observed for samples in dark (Figure 7a,b). To verify the generation of ∙O_2_^−^ and ∙OH, 5,5-dimethyl-1-pyrroline N-oxide (DMPO) were used as the indicator. Four peaks of equal intensity (1:1:1:1) were obviously observed for Ce_0.2_-BiOBr after 5 min of visible light irradiation, which was the characteristic signal of ∙O_2_^−^. However, the characteristic signal of O_2_^−^ was very weak in the spectrum of BiOBr. Therefore, more ∙O_2_^−^ could be generated by Ce_0.2_-BiOBr under visible light irradiation. Furthermore, the ∙OH signal with a peak intensity of 1:2:2:1 was very weak in the spectrum of Ce_0.2_-BiOBr, indicating that the concentration of ∙OH was quite low and might not play a critical role in the degradation of BPA.

To explore the mechanism of BPA photocatalytic degradation, Na_2_C_2_O_4_, t-butyl alcohol (TBA), ascorbic acid, and N_2_-purging were used to remove h^+^, ∙OH, and ∙O_2_^−^ and dissolved O_2_ during the degradation process, respectively. As is shown in Figure S7, these trapping experiments results show that the degradation efficiency decreased by 87%, 56%, 55%, and 10% in the presence of ascorbic acid, Na_2_C_2_O_4_, N_2_ purging, and TBA, respectively. The photocatalytic degradation of BPA was slightly affected by the addition of TBA, but strongly decreased with the addition of ascorbic acid, indicating that the contribution of each active species was in order of ∙O_2_^−^ > h^+^ >∙OH. Therefore, ∙O_2_^−^ was the main active species in the degradation process, and the contribution of h^+^ was also considerable. On the basis of the band structure of Ce_0.2_-BiOBr, the CBM was −0.39 eV, which was more negative than the reduction potential of O_2_/∙O_2_^−^ (−0.046 eV vs. NHE). And its VBM was +2.08 eV, which was less positive than the oxidation potential of OH^−^/∙OH (+2.38 eV vs NHE). This result confirms that the photogenerated e^−^ in the CB of Ce_0.2_-BiOBr could reduce the dissolved O_2_ to ∙O_2_^−^, but the photogenerated h^+^ in VB could not directly oxidize H_2_O to ∙OH. The ∙OH occurred during the degradation process could only indirectly generated through series of free radical reactions. The pathway of free radical generation was speculated to be as follows [38]:

Ce-BiOBr (or BiOBr) + *hv* → e^−^ + h^+^ (6)

e^−^ + O_2_ → ∙O_2_^−^ (7)

∙O_2_^−^ + H_2_O → ∙OOH + OH^−^ (8)

∙OOH + 2e^−^+ H_2_O → ∙OH + 2OH^−^ (9)

### 3.5. Theoretical Calculations

**Figure 8:**
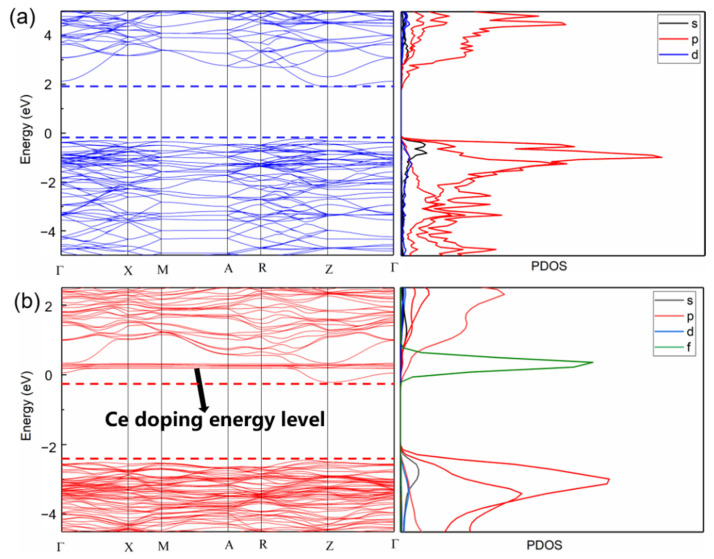
Electron band structure and state density diagram of (**a**) BiOBr and (**b**) Ce-BiOBr.

To determine the doping sites of Ce atoms in BiOBr crystals, the formation energies of Ce atoms in Bi, O, and Br sites were calculated. The optimized structure of BiOBr is shown in Figure S8a, and three types of Ce doping sites (Bi, O, and Br sites) are shown in Figure S8b,d. The formation energies of Ce atoms in Bi sites were lower than those in O and Br sites (Figure S9), indicating that Ce atoms tend to dope at the Bi site in the BiOBr crystal structure. In Figure S10, the calculated band gaps of Ce-BiOBr are 1.40 and 1.30 eV when Ce atoms dope at the Br and O sites, respectively. From the band structure and density of states (DOS) values shown in Figure 8, Ce doping had little effect on the band gap of BiOBr when Ce atoms doped at the Bi sites, which was more close to the previous experimental results. Ce doping caused the band of BiOBr to move to a lower energy direction, thus placing the Fermi level near the bottom of the CB. In addition, it is worth noting that an additional Ce-doping energy level was introduced in the CBM, which was mainly due to the contribution of the 4f e^−^ of Ce atoms. As a polyelectronic atom, Ce replaced some of the Bi atoms in the BiOBr crystal structure. The extra e^−^ only needed a low energy to be transferred to the CB, which could serve as a donor to provide e^−^, thereby increasing the number of e^−^ in the CB. This results in more e^−^ transferring to the surface to react with O_2_ to generate ∙O_2_^−^, which could enhance the degradation performance of the samples.

### 3.6. Reaction Model of the Photocatalytic Degradation of BPA over Ce-BiOBr Nanoflakes

**Figure 9:**
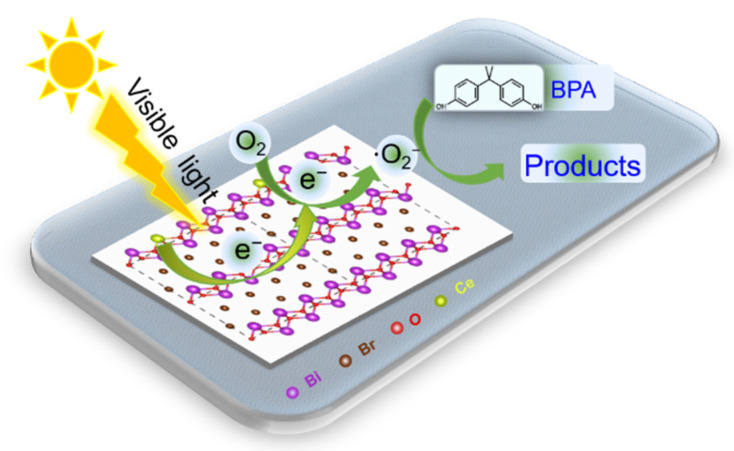
Schematic of BPA degradation over Ce-BiOBr nanoflakes.

A possible reaction model for the photocatalytic degradation of BPA over Ce-BiOBr nanoflakes is shown in Figure 9. On the one hand, the narrower band gap of Ce-BiOBr led to a stronger response to visible light. The photogenerated charge carriers in Ce-BiOBr possessed a longer lifetime, resulting in a greater generation of ∙O_2_^−^. On the other hand, the new Ce-doping energy level was introduced in the CB structure of BiOBr and increased the amount of e^−^ in the CB. Based on these results, more ∙O_2_^−^ was generated by the Ce_0.2_-BiOBr nanoflakes under visible light irradiation compared to BiOBr, which led to an enhanced BPA degradation efficiency under visible light irradiation.

## 4. Conclusions

In summary, Ce-BiOBr nanoflakes of uniform size were synthesized via a mild hydrothermal procedure. The Ce_0.2_-BiOBr nanoflakes possessed the highest photocatalytic activity for the degradation of BPA under visible light irradiation, which was 3.66 times higher than that of BiOBr. The enhanced photocatalytic activity could be ascribed to the narrowed band gap, enhanced charge carries separation efficiency and the Ce-doping energy level. Firstly, the doped Ce element reduced the band gap of the BiOBr and thus improved the visible light adsorption efficiency. Secondly, Ce_0.2_-BiOBr possessed a superior separation efficiency and longer lifetime of the charge carries than those of BiOBr, indicating that more photogenerated charge carriers could be injected into adsorbed molecules rather than recombined in the host crystal. Finally, based on the results of DFT calculation, the Ce-doping energy level was induced into the CB of BiOBr, which led to a higher amount of e^−^ in the CB. Therefore, more e^−^ were injected into the adsorbed O_2_ molecules and more O_2_^−^ were generated under visible light irradiation, which was the main active species for the degradation of BPA. In this way, this study developed an effective strategy for the modification of BiOBr and demonstrated its practical applications for water and wastewater treatment.

## Figures and Tables

**Table 1:** PL Lifetime Fitting Parameters for BiOBr and Ce_0.2_-BiOBr.

Sample	*B*_1_ (a.u.)	$\tau_{1}\;(\mathrm{ns})$	*B*_2_ (a.u.)	$\tau_{2}\;(\mathrm{ns})$
BiOBr	562.68	3.79	199.95	47.85
Ce_0.2_-BiOBr	560.90	4.68	228.19	52.37

## Data Availability

Not applicable.

## Supplementary Materials

The following supporting information can be downloaded at: https://www.mdpi.com/article/10.3390/nano12081382/s1, Figure S1: XPS survey spectra of BiOBr and Ce-BiOBr samples; Figure S2: O 1s XPS spectra of the (a) all samples, (b) BiOBr, (c) Ce_0.05_-BiOBr, (d) Ce_0.1_-BiOBr, (e) Ce_0.2_-BiOBr and (f) Ce_0.5_-BiOBr; Figure S3: N_2_ adsorption-desorption isotherms of BiOBr and Ce-BiOBr samples; Figure S4: Recycling properties of the photocatalytic degradation of BPA over Ce_0.2_-BiOBr nanosheets; Figure. S5: Zeta potential distribution of Ce_0.2_-BiOBr before and after five cycles of BPA degradation. Figure. S6: (a) TEM and (b) HRTEM images of Ce_0.2_-BiOBr after five cycles of BPA degradation. Figure S7: Photocatalytic degradation kinetic constants of BPA with added scavengers. Figure S8: Optimized structure of BiOBr, Ce doping on (b) Bi sites and (c) O sites and (d) Br sites. Figure S9: The formation energies of Ce atoms in Bi, Br, O sites, respectively. Figure S10: Electron band structure and state density diagram of Ce atoms in (a) Br and (b) O sites. Table S1: BET surface areas, pore volume and pore size of Ce_0.2_-BiOBr before and after five cycles of BPA degradation.
