# Supplementary material for: Electron-Level Mechanistic Insights into Ce Doping for Enhanced Efficiency Degradation of Bisphenol A under Visible Light Irradiation
Source: Nanomaterials (Basel). 2022 Apr 18;12(8):1382. doi: 10.3390/nano12081382 (PMC9027963; doi:10.3390/nano12081382)
Supplement: Supplementary file 1 [file nanomaterials-12-01382-s001.zip › nanomaterials-1661535-supplementary.pdf]

Supplementary Materials

Electron-Level Mechanistic Insights into Ce Doping for Enhanced Efficiency Degradation of Bisphenol A under Visible Light Irradiation

Qi Zeng †, Chu-Ya Wang *,†, Bo-Xing Xu, Jianyu Han, Xin Fang and Guangcan Zhu *

School of Energy and Environment, Southeast University, Nanjing 210096, China; zeng_qi@seu.edu.cn (Q.Z.); 213190216@seu.edu.cn (B.-X.X.); hanjy@seu.edu.cn (J.H.); fxin@seu.edu.cn (X.F.)

* Correspondence: wang-cy@seu.edu.cn (C.-Y.W.); gc-zhu@seu.edu.cn (G.Z.); Tel.: +86-17775346164 (C.-Y.W.); +86-18912966396 (G.Z.)

† These authors contributed equally to this work.

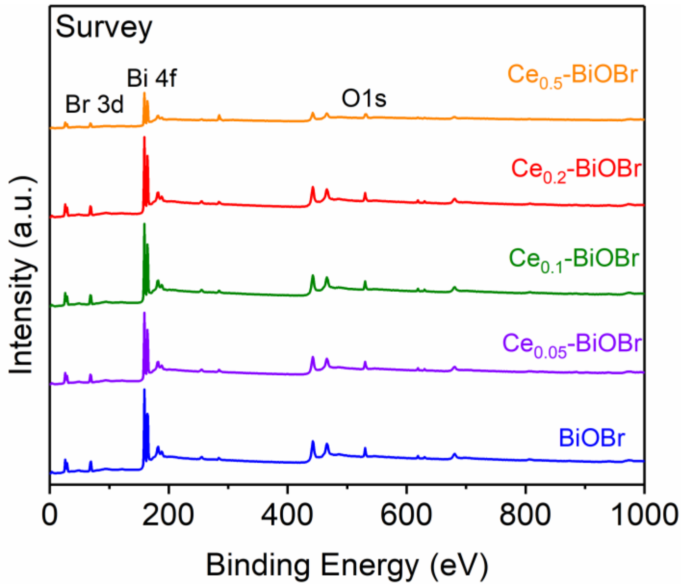

Figure. S1. XPS survey spectra of BiOBr and Ce-BiOBr samples.

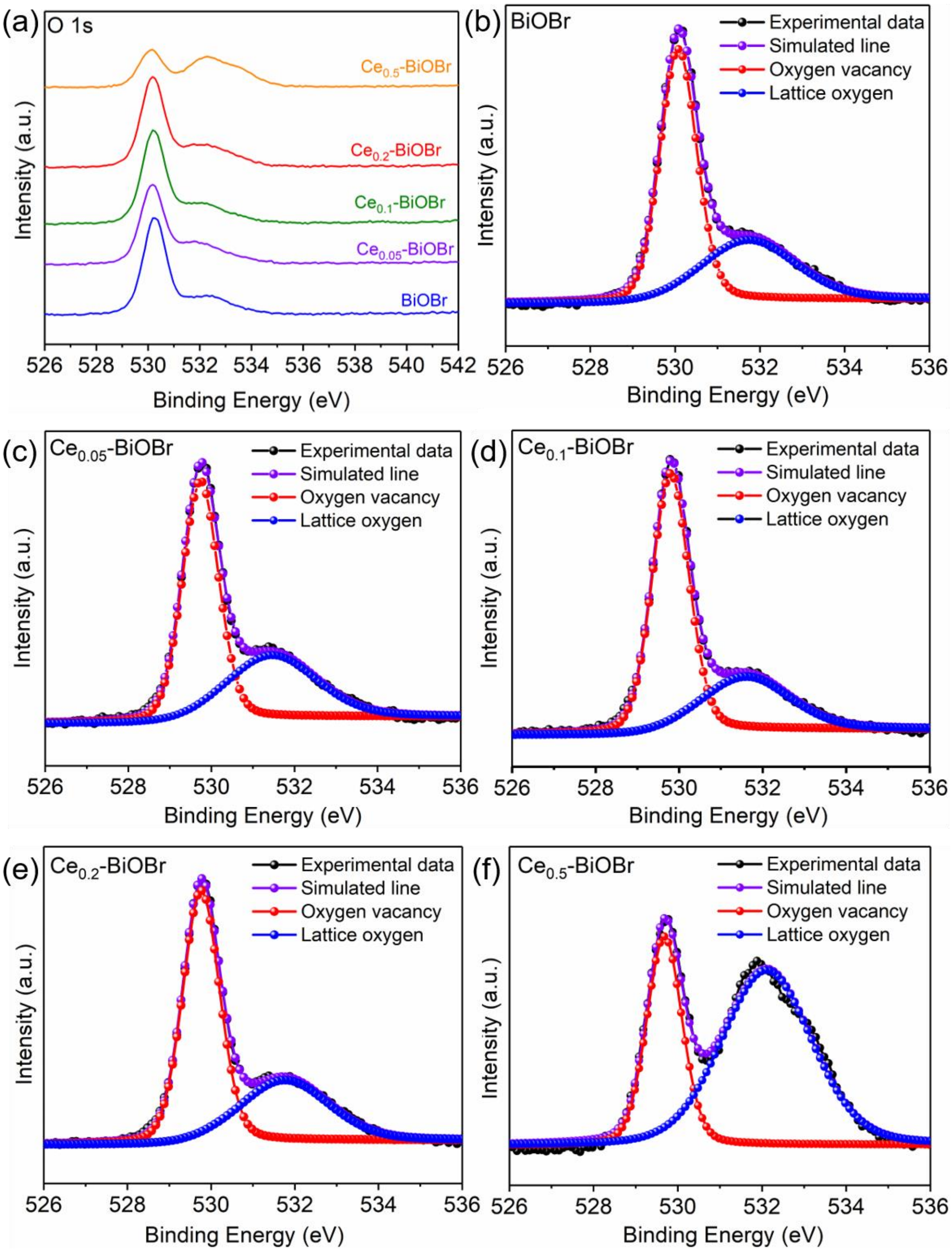

Figure. S2. O 1s XPS spectra of the (a) all samples, (b) BiOBr, (c) $Ce_{0.05}$-BiOBr, (d) $Ce_{0.1}$-BiOBr, (e) $Ce_{0.2}$-BiOBr and (f) $Ce_{0.5}$-BiOBr.

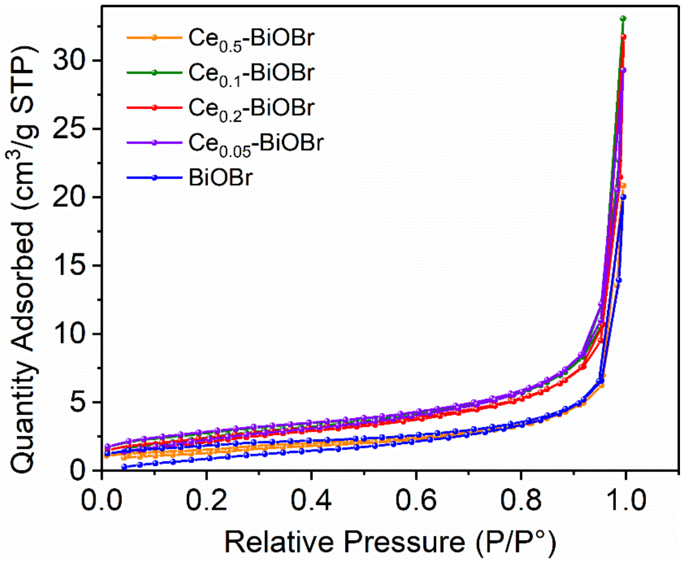

Figure. S3. N_2 adsorption-desorption isotherms of BiOBr and Ce-BiOBr samples.

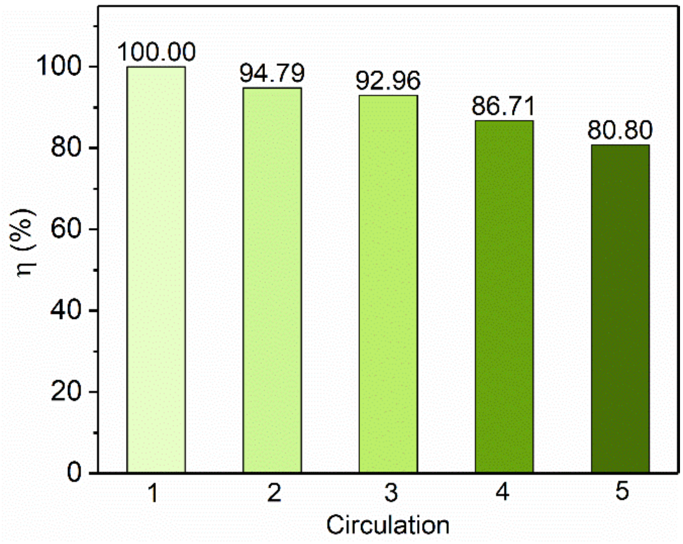

Figure. S4. Recycling properties of the photocatalytic degradation of BPA over $Ce_{0.2}$-BiOBr nanosheets.

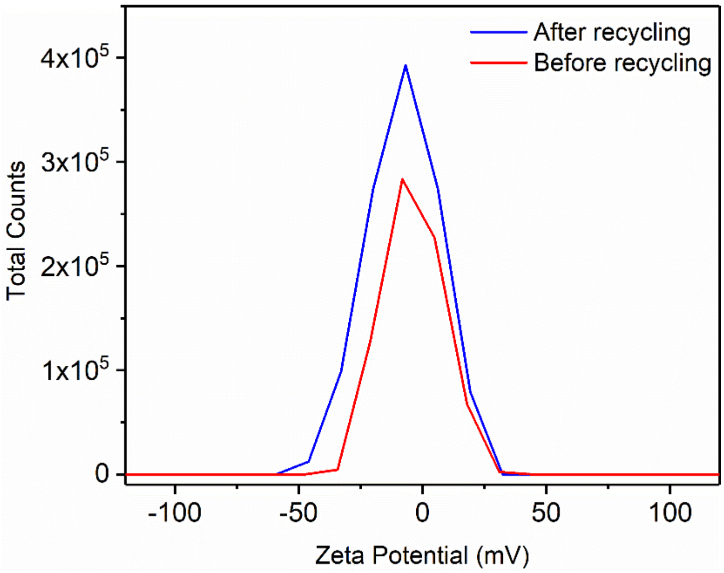

Figure. S5. Zeta potential distribution of $Ce_{0.2}$-BiOBr before and after five cycles of BPA degradation.

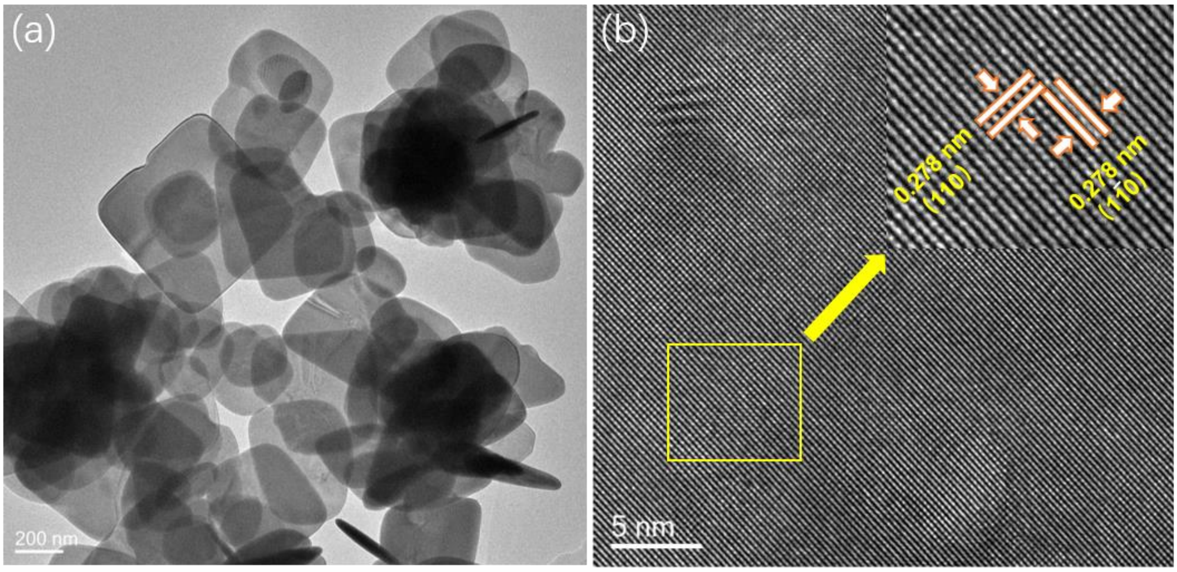

Figure. S6. (a) TEM and (b) HRTEM images of $Ce_{0.2}$-BiOBr after five cycles of BPA degradation.

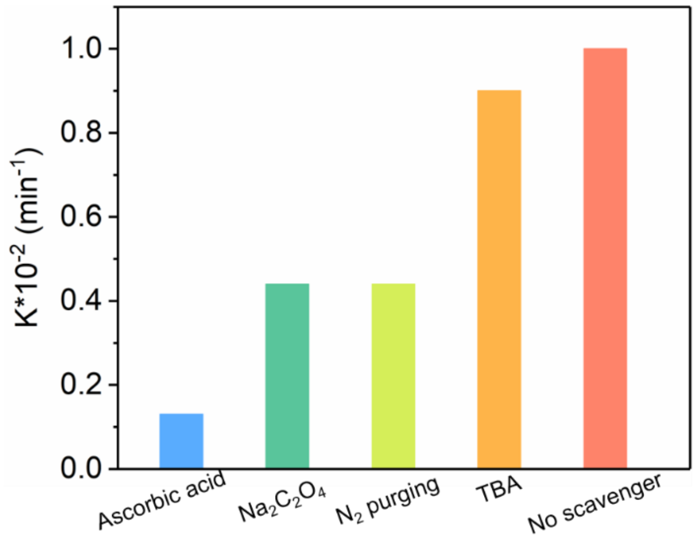

Figure. S7. Photocatalytic degradation kinetic constants of BPA with added scavengers.

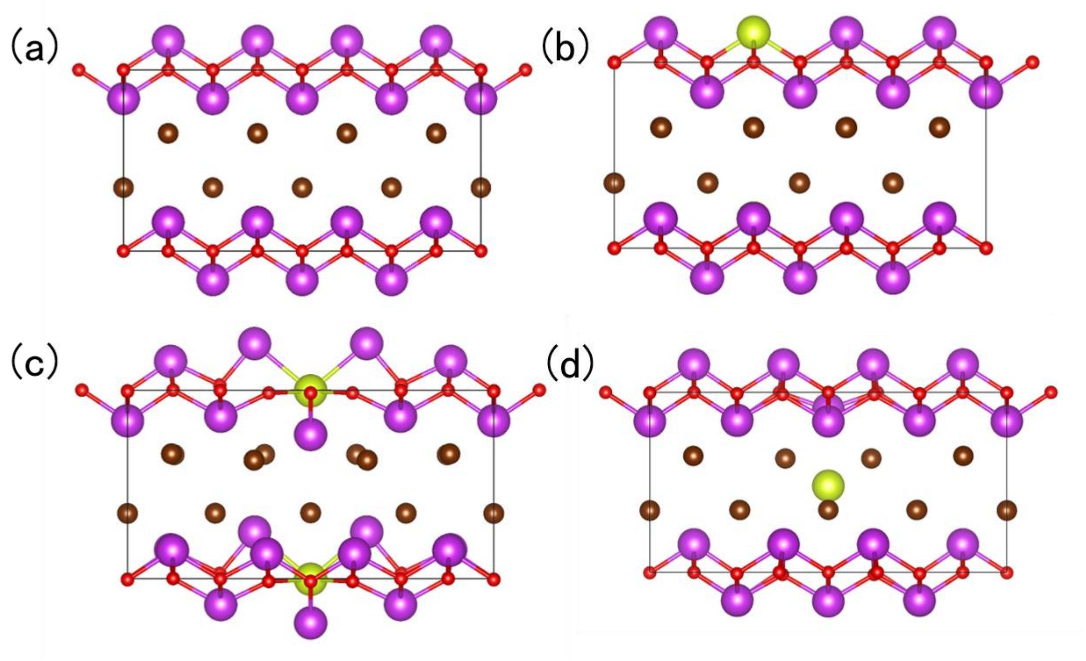

Figure. S8. (a) Optimized structure of BiOBr, Ce doping on (b) Bi sites and (c) O sites and (d) Br sites.

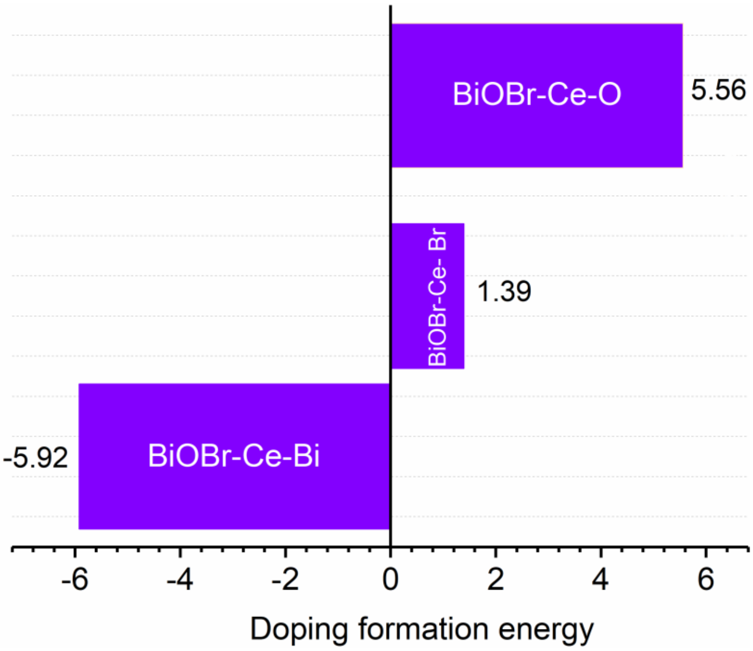

Figure. S9. The formation energies of Ce atoms in Bi, Br, O sites, respectively.

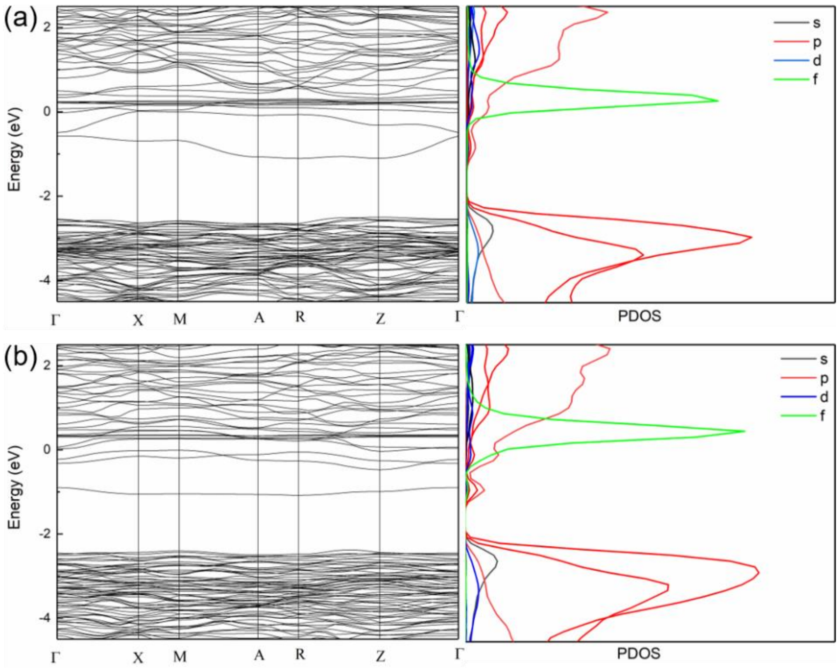

Figure. S10. Electron band structure and state density diagram of Ce atoms in (a) Br and (b) O sites.

Table. S1. BET surface areas, pore volume and pore size of $Ce_{0.2}$-BiOBr before and after five cycles of BPA degradation.

	S_{BET} ($m^2 \cdot g^{-1}$)	V_{pore} ($cm^3 \cdot g^{-1}$)	D_{pore} (nm)
**Before recycling**	8.55	0.049	22.94
**After recycling**	8.31	0.031	14.75
